# Respondents’ report of a clinician-diagnosed depression in health surveys: comparison with DSM-IV mental disorders in the general adult population in Germany

**DOI:** 10.1186/s12888-017-1203-8

**Published:** 2017-01-23

**Authors:** Ulrike E. Maske, Ulfert Hapke, Steffi G. Riedel-Heller, Markus A. Busch, Ronald C. Kessler

**Affiliations:** 1 Department of Epidemiology and Health Monitoring, Division 26 Mental Health, Robert Koch Institute, Berlin, Germany; 2 Institute of Social Medicine, Occupational Health and Public Health (ISAP), Faculty of Medicine, University of Leipzig, Leipzig, Germany; 3 Department of Health Care Policy, Harvard University, Boston, MA USA

**Keywords:** Depression, Diagnosis, Composite International Diagnostic Interview, General population, Health surveys

## Abstract

**Background:**

Respondents’ report of a previously diagnosed depression by a health professional is frequently used to estimate depression prevalence. This study contributes to a better understanding of survey results based on this measure by comparing it with a comprehensive standardized diagnostic interview.

**Methods:**

Data came from the cross-sectional nationwide German Health Interview and Examination Survey for Adults (DEGS1) and its mental health module (DEGS1-MH, *n* = 4483). In DEGS1, participants were asked whether they have been diagnosed with depression by a physician or psychotherapist (last 12-month). DSM-IV-based 12-month major depressive disorder (MDD) and other mental disorders were assessed with the German version of the Composite International Diagnostic Interview (CIDI). Time lag between both assessments was 6 weeks (median).

**Results:**

73.4% of participants reporting clinician-diagnosed depression met criteria for any mental disorder in the CIDI (any affective disorder: 51.8%, any anxiety disorder: 54.7%). The proportion of participants reporting a clinician-diagnosed depression who met MDD criteria was highest among those aged 18–29 years (62.6%) and decreased with age (65–79 years: 29.8%). Among participants with MDD, the proportion with clinician-diagnosed depression was 33.0%, highest among those aged 45–64 years (49.3%) and lowest among those aged 18–29 years (22.7%) and 30–44 years (20.3%). MDD severity was positively associated with clinician-diagnosed depression.

**Conclusions:**

Respondents’ report of a clinician-diagnosed depression and major depression assessed with the CIDI substantially differ. Concordance of both measures varies with age and severity of depressive symptoms. Health surveys should assess a range of depression indicators in order to cover a wide spectrum.

**Electronic supplementary material:**

The online version of this article (doi:10.1186/s12888-017-1203-8) contains supplementary material, which is available to authorized users.

## Background

In many large-scale health surveys, the prevalence of depression is assessed by asking participants whether they have been diagnosed with depression by a health professional in the past 12 months [1–4]. This simple measure has various preconditions such as previous health care utilization of the respondent, reporting depressive symptoms to a health professional, understanding the question asked in the survey, and admitting the diagnosis in the survey interview. In addition, the accuracy of this measure in determining the existence of depression depends on whether health professionals correctly diagnose their patient with depression. The latter can be at least partly questioned considering the notable extent of over- and under-diagnosis of depression in primary care found in clinical and community studies [5–8].

Despite these preconditions, a recent general population survey found that 12-month prevalence estimates based on the respondent’s report of a clinician-diagnosis of depression and an interviewer assessment of major depression with a comprehensive standardized diagnostic interview based on the Diagnostic and Statistical Manual of Mental Disorders-IV (DSM-IV) were on a similar level [1]. Yet, the overlap of the two measures was only moderate, and, in agreement with previous research [2, 4, 9, 10], prevalence of major depression was highest in younger age adults and of clinician-diagnosed depression in middle-aged and older adults [1]. These findings suggest that there are substantial differences between DSM-IV-based major depression and a clinician-diagnosed depression and, as a consequence, that the presence of major depression may not be inferred with accuracy from the respondent’s report of a previous clinician diagnosis. However, there is a lack of specific knowledge about differences between both measures at an individual level.

Considering the fact that depression is still quite often assessed with a single question about clinician diagnoses in health surveys, this study aims to contribute to a better understanding of survey results based on the respondent’s report of a clinician-diagnosed depression. Therefore, we investigate the association between such reports and independent diagnoses of depression in a large general population survey. Specifically, we examine the proportion of mental disorders based on a comprehensive standardized diagnostic research interview among survey participants who report a clinician-diagnosed depression as a function of sex, age, and depression severity. Moreover, in order to examine the false negative rate based on the question about a previous clinician diagnosis, we examine the proportion of survey participants with major depression determined by the diagnostic interview who do versus do not report that a clinician diagnosed them with depression. Finally, socio-demographic, health-related and mental health characteristics are reported separately for survey participants with different combinations of depression diagnoses based on the cross-classification of diagnoses according to a diagnostic interview administered in the survey and the respondents’ reports of being diagnosed by a clinician.

## Methods

### Study design and sample

Data come from the cross-sectional nationwide “German Health Interview and Examination Survey for Adults” (DEGS1) and its mental health module (DEGS1-MH; *n* = 4483; age 18–79 years). The design, objectives and methods have been described in detail elsewhere [11–14]. Briefly, a random sample of persons aged 18–79 years stratified for sex, age and geographical location was selected using two-stage clustered random sampling. On the first stage, 180 sample points were drawn from all German municipal communities, on the second stage, participants were randomly drawn from population registries of these sample points. For DEGS1-MH, all DEGS1 participants with complete assessment (interview and examinations) aged 18–79 years were eligible who had consented to being re-contacted for the mental health module, who had sufficient language skills and who were available during the assessment period [11, 14]. In DEGS1, data were collected by self-administered written questionnaire and a standardized physician-administered computer-assisted personal interview (CAPI) and physical and laboratory measurements. In DEGS1-MH, 12-month and lifetime diagnoses of mental disorders based on the diagnostic criteria of the DSM-IV-TR were assessed in a CAPI using a modified version of the Composite International Diagnostic Interview (DIA-X/M-CIDI) [15, 16], a German version of the internationally established CIDI [15, 17, 18]. The median time lag between DEGS1 and DEGS1-MH was 6 weeks (inter quartile range 5–25 weeks). DEGS1 was approved by the federal and state commissioners for data protection and by the ethics committee of Charité-Universitätsmedizin Berlin (No.EA2/047/08). DEGS1-MH was additionally approved by the ethics committee of the Technische Universität Dresden (No.EK174062009). All participants provided written informed consent.

### Measures

#### Clinician-diagnosed depression based on the respondents’ report

Lifetime clinician-diagnosed depression was assessed in the physician-administered CAPI in DEGS1, where participants were asked: “Have you ever been diagnosed with depression by a physician or a psychotherapist?” If affirmed, 12-month clinician-diagnosed depression was determined with the question: “Was the depression present during the last 12 months?”

#### Major depressive disorder (MDD) and other mental disorders based on a diagnostic interview

MDD in the past 12 months was determined by the DIA-X/M-CIDI. In the DIA-X/M-CIDI, the presence of depression symptoms is assessed in 30 single items, which are grouped into the nine depression symptoms of DSM-IV based on a standardized algorithm. MDD was determined by applying all DSM-IV diagnostic criteria. Compared to a clinical lifetime diagnosis, a sensitivity of 0.95 (single depressive episode, lifetime) and 0.929 (recurrent major depressive episode, lifetime), and a specificity of 1.0 for both were reported [19]. In the same study, Kappa values of 0.82 for single major depression and 0.9 for recurrent major depression were found for the agreement of 1-month CIDI-based diagnosis and clinician-diagnosis. Substantial test-retest reliability has been shown for the M-CIDI [15].

Further 12-month mental disorders were assessed by the DIA-X/M-CIDI: other mood disorders (dysthymia, bipolar disorder I and II), anxiety disorders (panic disorder, agoraphobia, generalized anxiety disorder, social phobia, specific phobias, obsessive-compulsive disorder, post-traumatic stress disorder (PTSD)), somatoform disorders (pain disorder, undifferentiated somatoform disorder measured with the Somatic Symptom Index, SSI4,6; [20]), substance use disorders (alcohol and medication/drug abuse and dependence) without nicotine dependence, possible psychotic disorders (screening without further differential diagnosis), eating disorders (anorexia nervosa, bulimia nervosa, binge eating disorder) and mental disorder due to general medical conditions or substance induced disorders.

#### Depression severity

In addition to the presence of any comorbid mental disorder as described above excluding MDD, the following depression severity indicators were defined. For participants with MDD, the number of depression symptoms affirmed in the CIDI depression section was categorized into the following groups taking the lower and upper quartile as cut-offs: 5 symptoms (mild depression), 6–7 symptoms (moderate depression) and 8–9 symptoms (severe depression). Moreover, affirmation of thoughts about death or suicidal plan or attempt was established as a second CIDI-based severity indicator. Psychiatric comorbidity was determined for all participants based on all mental disorders assessed excluding MDD. Based on the self-administered German version of the Patient Health Questionnaire-9 (PHQ-9) [21, 22], which was assessed in all participants in DEGS1 and in DEGS1-MH, two additional severity indicators were defined: first, having depressive symptoms in both PHQ-9 assessments (i.e. sum score of 10 or more points in DEGS1 and DEGS1-MH) [21, 23] and second, having reported thoughts of being better off dead or of hurting oneself for at least several days in both PHQ-9 assessments.

#### Socio-demographic and health related characteristics

In DEGS1, sex, age, marital status, employment status and a range of further socio-demographic and health-related characteristics were assessed. Number of outpatient physician visits (excluding dentist visits) in the past 12 months was determined in a self-administered questionnaire based on a comprehensive list of medical disciplines. Including medical psychotherapist and psychological psychotherapist visits, the number of outpatient visits (minimum 0, maximum 155) was categorized into quartiles (0–2, 3–5, 6–9, ≥10) for analyses. Education was grouped into low, middle and high based on the Comparative Analysis of Social Mobility in Industrial Nations (CASMIN) [24]. Socio-economic status (SES) was classified as low, middle and high using an index based on information on education, occupational status and net household income [25]. Self-perceived social support was assessed using the Oslo-3 Social Support Scale and categorized as poor (3–8 points), moderate (9–11 points) and strong support (12–14 points) [26]. Community size was determined based on official administrative municipal codes for the place of residence. Health-related quality of life was examined using the physical and mental component scores of the Short Form 36 (SF-36) [27, 28].

### Statistical analyses

Proportions are reported with 95% confidence intervals (95%CI). Pearson’s *χ* ^2^ test was used to determine whether two categorical variables were independent on a significance level of 0.05. Associations were determined using bivariate and multivariate logistic regression models. To test for trend over the age groups, age group was included in the logistic regression model as a continuous variable. To analyse whether time lag between DEGS1 and DEGS1-MH has an influence on the results, the following analyses were carried out: Sensitivity analyses were conducted by excluding the upper quartile from the analyses (time lag to >25 weeks). Additionally, cross-tables showing the overlap of CIDI-based MDD and clinician-diagnosed depression stratified for the time lag (0–25 weeks vs. more than 25 weeks) can be found in the Additional file 1. Further, a logistic regression model with CIDI-based MDD as dependent variable and clinician-diagnosed depression as independent variable was calculated, including an interaction term of clinician-diagnosed depression and time lag (in quartiles). The interaction term was not significant (*p* = 0.152).

Analyses were conducted with a weighting factor which 1) accounts for the complex sampling design (selection probability of the sample point and selection probability of the respondent within the sample point) and 2) corrects sample deviations from the population structure as of 31 Dec 2010 with regard to age group, sex, region, nationality, community type, education and participation in DEGS1-MH [11, 14, 29]. Confidence intervals were determined using STATA’s survey procedures (STATA 14), which takes into account the weighting and the correlation of participants within a sample point. A non-responder analysis and a comparison with data from official statistics indicate that the DEGS1 sample is highly representative of the population aged 18–79 years in Germany [29].

## Results

Of *n* = 4483 participants of DEGS1-MH, *n* = 101 (2.3%) had missing values in at least one of both measures, resulting in a study sample of *n* = 4382. In the sample, 50.9% (95%CI 48.9–52.9) were female, 19.2% (95%CI 17.9–20.5) were 18–29 years old, 24.9% (95%CI 23.3–26.6) were 30–44 years old, 36.0% (95%CI 34.2–37.8) were 45–64 years old and 20.0% (95%CI 18.7–21.3) were 65–79 years old. Overall, there were 78.8% (95%CI 76.8–80.7) married and living with the partner or in a steady relationship. The distribution of CASMIN educational level was 35.0% (95%CI 32.5–37.5) for low, 50.8% (95%CI 48.7–52.9) for middle and 14.2% (95%CI 12.6–16.0) for high.

### Mental disorders among participants with a clinician-diagnosed depression

Among those reporting a clinician-diagnosed depression, 73.4% had any mental disorder, of which anxiety disorders and affective disorders were the most frequent ones (Table 1). The proportion of all mental disorders was higher in participants with a clinician-diagnosed depression than in participants without a clinician-diagnosed depression in the past 12 months.

**Table 1:** Proportion of mental disorders among participants with and without a clinician-diagnosed depression

	12-month clinician-diagnosed depression (*n* = 249)	No 12-month clinician-diagnosed depression (*n* = 4133)	*p*
12-month mental disorders	%(w) (95%CI)	%(w) (95%CI)	
Any anxiety disorder^a^	54.7 (46.0–63.1)	16.8 (15.2–18.6)	<0.0001
Any affective disorder^b^	51.8 (42.9–60.5)	7.4 (6.4–8.5)	<0.0001
Possible psychotic disorder (screening)	17.3 (11.2–25.9)	2.0 (1.5–2.6)	<0.0001
Any substance use disorder^c^	15.2 (10.7–21.2)	5.4 (4.4–6.5)	<0.0001
Any somatoform disorder^d^	14.8 (10.4–20.7)	3.0 (2.4–3.8)	<0.0001
Any GMC/substance induced disorder	7.7 (3.4–16.4)	0.8 (0.5–1.2)	<0.0001
Any eating disorder^e^	3.5 (1.0–11.5)	0.8 (0.6–1.3)	0.0227
Any of the above	73.4 (64.2–80.9)	26.0 (24.1–28.0)	<0.0001

^a^ including panic disorder, agoraphobia, generalized anxiety disorder, social phobia, specific phobias, obsessive-compulsive disorder, PTSD

^b^ including major depression, dysthymia, bipolar disorder I or II

^c^ including alcohol and medication abuse and dependence

^d^ pain disorder and undifferentiated somatoform disorder as measured by the Somatic Symptom Index, SSI4,6 (Escobar et al., 1989)

^e^ including anorexia nervosa, bulimia nervosa, binge eating disorder

### MDD among participants with a clinician-diagnosed depression

**Fig. 1:**
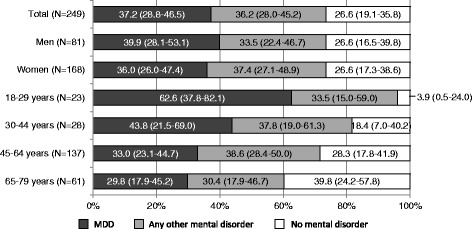
Cumulative proportions of 12-month major depressive disorder (MDD) and any other mental disorder^1^ among participants with clinician-diagnosed depression in the past 12 months. ^1^Including dysthymia, bipolar disorder I and II, panic disorder, agoraphobia, generalized anxiety disorder, social phobia, specific phobias, obsessive-compulsive disorder, PTSD, pain disorder, undifferentiated somatoform disorder, alcohol and medication abuse and dependence, possible psychotic disorders (screening without further differential diagnosis), anorexia nervosa, bulimia nervosa, binge eating disorder, mental disorder due to general medical conditions or substance induced disorders.

Among participants with a clinician-diagnosed depression, no substantial differences in the proportion of MDD and of any other mental disorder were found between men and women (Fig. 1). The percentage of participants with a clinician-diagnosed depression who also met the criteria of MDD was 62.6% in the age group of 18–29 years (and 3.9% remained without any mental disorder) and decreased to 29.8% in the age groups of 65–79 years (and 39.8% remained without any mental disorder). The odds of meeting the criteria of MDD when reporting a clinician-diagnosed depression decreased per age group (OR: 0.6, 95%CI 0.4–0.9; p^trend^ = 0.021). The vast majority of those without clinician-diagnosed depression did not fulfil the criteria of MDD (men: 97.3%, 95%CI 96.3–98.0; women: 92.7%, 95%CI 90.9–94.2; 18–29 years: 92.2%, 95%CI: 88.8–94.6; 30–44 years: 93.1%, 95%CI 90.1–95.2; 45–64 years: 96.7%, 95%CI 95.6–97.5; 65–79 years: 97.3, 95%CI 95.5–98.4).

Among participants with a clinician-diagnosed depression, meeting the criteria of MDD was more likely with any other mental disorder present and with current depressive symptoms and the suicide item affirmed in both PHQ-9 assessments (Table 2).

**Table 2:** Proportion of major depressive disorder (MDD) among participants with clinician-diagnosed depression across depression severity indicators

	Participants with a clinician-diagnosed depression in the past 12 months (*n* = 249)					
	12-month MDD	No 12-month MDD				
	Row %(w) (95%CI)	Row %(w) (95%CI)	Bivariate OR (95%CI)	*p*	Adjusted OR^a^(95% CI)	*p*
Any comorbid mental disorder^b^ (*N* = 171)	48.0 (37.0–59.2)	52.0 (40.8–63.0)	6.4 (2.4–17.2)	<0.001	5.8 (2.2–15.3)	<0.001
No comorbid mental disorder (*N* = 78)	12.6 (5.9–25.0)	87.4 (75.0–94.1)	Ref.		Ref.	
Current depressive symptoms^c^(*N* = 64)	63.4 (46.9–77.3)	36.6 (22.7–53.1)	4.6 (2.3–9.5)	<0.001	4.6 (2.2–9.5)	<0.001
No current depressive symptoms (*N* = 185)	27.2 (19.5–36.6)	72.8 (63.4–80.5)	Ref.		Ref.	
Affirmed suicide item^d^ (*N* = 52)	63.9 (45.5–79.0)	36.1 (21.0–54.5)	4.2 (1.7–10.0)	0.002	4.1 (1.7–10.1)	0.002
Suicide item not affirmed (*N* = 197)	29.8 (21.4–40.0)	70.2 (60.0–78.6)	Ref.		Ref.	

^a^ adjusted for sex and age

^b^ dysthymia, bipolar disorder I and II, panic disorder, agoraphobia, generalized anxiety disorder, social phobia, specific phobias, obsessive-compulsive disorder, PTSD, pain disorder, undifferentiated somatoform disorder, alcohol and medication abuse and dependence, possible psychotic disorders (screening without further differential diagnosis), anorexia nervosa, bulimia nervosa, binge eating disorder, mental disorder due to general medical conditions or substance induced disorders

^c^ PHQ-9 current depressive symptoms (sum score ≥10) in DEGS1 and DEGS1-MH

^d^ PHQ-9 suicide item affirmed (at least several days) in DEGS1 and DEGS1-MH

### Clinician-diagnosed depression among participants with MDD

Among participants with MDD, the proportion of a clinician-diagnosed depression did not differ between men and women, but it was lower in participants of younger than of older age (Table 3). Among those without MDD, the vast majority did not report a clinician-diagnosed depression.

**Table 3:** Proportion of participants with and without clinician-diagnosed depression among participants with and without major depressive disorder (MDD)

	12-month clinician-diagnosed depression in participants with 12-month MDD	No 12-month clinician-diagnosed depression in participants without 12-month MDD
	%(w) (95%CI)	%(w) (95%CI)
Total	33.0 (25.9–40.9)	95.8 (94.8–96.6)
Sex		
Men	37.1 (26.0–49.7)	97.6 (96.6–98.3)
Women	31.3 (22.9–41.1)	94.0 (92.1–95.5)
Age group (years)		
18–29 years	22.7 (12.4–37.9)	98.5 (96.8–99.3)
30–44 years	20.3 (8.8–40.3)	97.6 (96.1–98.6)
45–64 years	49.3 (38.3–60.4)	93.6 (91.4–95.4)
65–79 years	44.2 (26.5–63.5)	95.0 (92.7–96.6)

Among participants with MDD, clinician-diagnosed depression was less often reported by those aged 18–29 years (OR: 0.3, 95%CI 0.1–0.7; *p* = 0.004) and 30–44 years (OR: 0.3, 95%CI 0.1–0.8; *p* = 0.014) compared to the age group of 45–64 years. When adjusting for sex and the number of outpatient physician visits, these differences between the age groups, i.e. the different proportion of clinician-diagnosed depression among those with MDD, did not remain significant. In those aged 65–79 years with MDD, clinician-diagnosed depression was reported as often as by those aged 45–64 years (0.8, 95%CI 0.3–1.9; *p* = 0.641).

Among those with MDD, participants with any comorbid mental disorder and with severe MDD were more likely of reporting clinician-diagnosed depression (Table 4).

**Table 4:** Proportion of clinician-diagnosed depression among participants with major depressive disorder (MDD) across depression severity indicators

	Participants who met the criteria for 12-month MDD (*n* = 284)					
	12-month clinician-diagnosed depression	No 12-month clinician-diagnosed depression				
	Row %(w) (95%CI)	Row %(w) (95%CI)	Bivariate OR (95%CI)	*p*	Adjusted OR^a^ (95% CI)	*p*
Any comorbid mental disorder^b^	37.9 (29.4–47.3)	62.1 (52.7–70.6)	3.3 (1.3–8.5)	0.012	6.6 (1.7–25.6)	0.006
No comorbid mental disorder^b^	15.5 (7.4–29.8)	84.5 (70.2–92.6)	Ref.		Ref.	
Number of depression symptoms						
Mild (5/9 symptoms)	19.0 (10.5–31.9)	81.0 (68.1–89.5)	Ref.		Ref.	
Moderate (6&7/9 symptoms)	27.2 (19.4–36.7	72.8 (63.3–80.6)	1.6 (0.7–3.7)	0.275	2.3 (0.9–5.4)	0.066
Severe (8&9/9 symptoms)	49.5 (34.4–64.7)	50.5 (35.3–65.6)	4.2 (1.7–10.4)	0.002	7.0 (2.6–19.0)	<0.001
Thoughts of death or suicide, or having suicide plan or attempt^c^	41.8 (32.3–52.0)	58.2 (48.0–67.7)	3.6 (1.8–7.3)	<0.001	3.2 (1.4–7.4)	0.006
No thoughts of death or suicide, or having suicide plan or attempt^c^	16.5 (10.2–25.6)	83.5 (74.4–89.8)	Ref.		Ref.	

^a^ adjusted for sex, age groups, number of outpatient physician visits

^b^ dysthymia, bipolar disorder I and II, panic disorder, agoraphobia, generalized anxiety disorder, social phobia, specific phobias, obsessive-compulsive disorder, PTSD, pain disorder, undifferentiated somatoform disorder, alcohol and medication abuse and dependence, possible psychotic disorders (screening without further differential diagnosis), anorexia nervosa, bulimia nervosa, binge eating disorder, mental disorder due to general medical conditions or substance induced disorders

^c^ based on the CIDI depression section

The only depression symptom associated with higher odds of reporting a clinician-diagnosed depression was “thoughts of death or suicide, or having suicide plan or attempt” (results for other symptoms not shown).

### Socio-demographic, health-related and mental health characteristics of participants with a clinician-diagnosed depression and MDD, with a clinician-diagnosed depression only and MDD only

Further analyses found significant overall differences of each socio-demographic characteristic considered between participants with clinician-diagnosed depression and MDD, with clinician-diagnosed depression only, with MDD only, and the non-cases according to both instruments (see Additional file 2). Participants with clinician-diagnosed depression who also met the criteria of MDD had the least favourable socio-demographic distributions, e.g. highest percentage of unmarried and without steady relationship, low SES, poor social support, never employed. Participants with clinician-diagnosed depression with and without MDD had higher numbers of outpatient physician visits and of chronic somatic conditions and a lower physical health related quality of life than participants with MDD only. Mental health related quality of life was lowest among participants with clinician-diagnosed depression and MDD.

The proportion of mental disorders was the highest in participants with clinician-diagnosed depression who also met the criteria of MDD than in participants with a clinician-diagnosed depression only or MDD only, though not significantly throughout the disorders examined (see Additional file 3). Compared to participants with a clinician-diagnosed depression only and MDD only, those identified by both measures had a higher proportion of PHQ-9 current depressive symptoms and of PHQ-9 suicidal item affirmed in DEGS1 and DEGS1-MH.

## Discussion

Based on a nationally representative sample of the general adult population, this study shows that a large proportion of survey participants who report a previously clinician-diagnosed depression meets criteria of at least one DSM-IV mental disorder. At the same time, there were major differences between participants reporting a clinician-diagnosed depression and participants who meet criteria for major depression in a comprehensive standardized diagnostic interview.

The findings of this study should be considered in the context of several limitations. **First**, the time lag between DEGS1 and DEGS1-MH might have led to an underestimation of the overlap of both measures examined and of the prevalence of mental disorders among participants with a clinician-diagnosed depression due to remission or to the time difference between both assessments. Analyses limiting the time lag to ≤25 weeks led to a slight increase of overlap, but the results did not substantially change. The time-lag stratified analyses of the overlap of both measures indicates that the time lag might have led to an age- or sex-specific under- or over-estimation of the overlap. However, the confidence intervals are large due to the small numbers of cases in the upper quartile. **Second**, the measure of a clinician-diagnosed depression is diffuse in two ways. The wording of the question leaves it unclear whether the depression diagnosis was made in the past 12 months or whether the respondent had received a diagnosis previously and interprets depressive symptoms in the past 12 months as a continuation. Further, the measure depends on the respondent’s ability to recall a depression diagnosis, which however also applies for symptom reports in the CIDI. **Third**, the mental disorders assessed in DEGS1-MH does not include all possible disorders, hence the prevalence of any other mental disorder might be underestimated. **Fourth**, the small number of cases results in large confidence intervals and restricts analyses with respect to the number of strata.

Despite these limitations, this study contributes to a better understanding of survey results based on the measure of a clinician-diagnosed depression. There are four noteworthy findings. **First**, there was a remarkable age pattern: Among participants with a clinician-diagnosed depression, the proportion of those meeting the criteria of MDD was 62.6% in those aged 18–29 years and decreased per age group to 29.8% in those aged 65–79 years. A similar yet less pronounced age pattern was found in a large community-based study in the US [5]. Interestingly, our results show that 96.1% of young adults who report a clinician-diagnosed depression had any mental disorder, while 39,8% of adults aged 65–79 years did not have any disorder even though reporting a health professional-diagnosed depression. The relatively high proportion of 62.6% MDD among those aged 18–29 years is higher than the 42.0% that was reported in a meta-analysis on the accuracy of unassisted depression diagnosis in primary care [30] and equal to the proportion of 66.5% which was reported for the optimal threshold for the CIDI screening scale [31]. Our study indicates that a clinician-diagnosed depression reported by this age group in a health survey might include to a large part participants who actually fulfilled the MDD criteria. A possible explanation for this finding is the higher prevalence of severe depression among depressed young adults [10] which might lead to more accurate diagnoses by health professionals. Also, health professionals might be more rigorous in diagnosing depression when it comes to younger patients because of its possible psychological impact on the life young people. Further, the high prevalence of depression in young adults [9, 10, 14, 32] might result in a higher number of “true positives” than in populations with lower depression prevalence. The high proportion of middle-aged and older participants with a clinician-diagnosed depression who did not meet the criteria of MDD implies a substantial amount of over-diagnosis of depression for these groups, replicating results from primary care in Italy [6] and the US [5]. Increased physical complaints in older age could be misinterpreted as symptoms of depression by health professionals [33, 34]. Additionally, underreporting of depressive symptoms by older participants in the CIDI due to its complex questioning and its multiple time frames [35] and due to problems recalling the symptoms are possible methodological explanations. Research has suggested that older adults more frequently show clinically significant depressive symptoms without fulfilling all diagnostic criteria [36]. In this context, our finding could at least partly be explained by the fact that the depression diagnosis based on the CIDI might exclude older people due to the diagnostic algorithm who might have reported significant symptoms to the health professional, resulting in a depression diagnosis.

The **second** notable result is the striking age pattern of the proportion of clinician-diagnosed depression among participants with MDD (low in young age groups and highest in those aged 45–64 years). Obviously, these findings cannot be interpreted as the percentage of actual unrecognition of depression by health professionals. Instead, it reflects the specific preconditions of the measure of a clinician-diagnosed depression reported by the respondent. Thus, our findings supposedly mirror to a large part the lack of any health professional contact in people with mood disorders [37] and the fact that young adults are less likely to use professional health services due to mental health problems [38–40] than older adults, but prefer seeking help rather from family or friends [41]. Additionally, low numbers of physician contacts are reported for younger adults [42], reducing the odds for a physician to detect depression [8, 43]. This is supported by the fact that the age pattern disappeared after controlling for the number of outpatient physician visits. In contrast to the US, where financial barriers have been reported the most common reasons for not seeking professional help for mental health problems [44], personal help-seeking barriers such as self- and perceived stigmatization [45–47] are likely more relevant in Germany, as diagnosis and treatment of mental disorders are covered by mandatory health insurance. As to depression recognition itself, our results reflect the considerable amount of unrecognized depression in primary care in Germany and internationally [8, 30, 48], which was suggested of being the most pronounced in younger people [7]. In addition, health professionals might not always fix and communicate a depression diagnosis to the patient [49]. Not least, self-stigmatization might lead to a reporting bias of a depression diagnosis in the survey interview.

**Third**, it is remarkable that no differences were found between men and women. On one hand, concerning the similar level of the proportion of MDD among men and women with a clinician-diagnosed depression, our results agree with findings from the US [5]. On the other hand, the fact that we did not find any sex difference regarding the proportion of clinician-diagnosed depression among those with MDD is contra-intuitive as research has suggested sex-specific barriers to help seeking [50], utilization of somatic and mental health services [42, 51], symptom reporting [52] and depression recognition rates [8].

**Fourth**, we found that participants with severe depression according to different severity indicators were more likely to be classified as cases by both measures. This is plausible considering that research found an association of seriousness of mental disorder with service use [38] and higher proportions of depression recognition in primary care when depression was severe and when suicidal ideation or tendency was reported by the patient [8]. This finding indicates that a considerable part of participants with severe depression is included in the measure of a clinician-diagnosed depression. In addition to the explanations given above, this finding can also be interpreted in the context of discussions about the DSM-IV potentially assigning a depression diagnosis partly to people without relevant disability [53]. Following this discourse, participants with depression without relevant disabilities might have qualified for a depression diagnosis in the CIDI, but were unlikely to seek for professional help due to their lack of disability.

## Conclusions

This study contributes to a better understanding of survey results based on the respondent’s report of a clinician-diagnosed depression. It shows that a large proportion of survey participants who report a clinician-diagnosed depression meets criteria of at least one DSM-IV mental disorder (most frequently any affective and any anxiety disorder) according to the CIDI. However, substantial differences exist between participants who report a clinician-diagnosed depression and participants who meet criteria for depression in a comprehensive standardized diagnostic interview. Furthermore, concordance of both measures varies with age and severity of depressive symptoms. Thus, respondent reports of clinician-diagnosed depression cannot be considered a short alternative for CIDI-based major depression in studies of the general population. This needs to be considered when interpreting survey results based on reports about clinician-diagnosed depression.

Instead, this study underlines the importance to assess a range of depression indicators in health surveys in order to cover a wide spectrum. For many research questions, information about depression diagnosis in the health care system is important. But if information on depressive symptoms or on the presence of diagnostic criteria is needed, further indicators such as standardized diagnostic interviews or one of the established short screening scales, which have been shown to have good concordance with diagnoses based on independent research diagnostic interviews [21, 31], should be additionally assessed.

Regarding clinical practice, our findings first imply that if patients report that they have not been diagnosed with depression, they most likely do neither have a history of major depression. Second, relating to younger patients, health professionals should be especially attentive and keep a possible major depression in mind when depressive symptoms are reported in order to provide adequate treatment. Third, relating to older patients, our results imply that health professionals should on one hand be more rigorous when diagnosing depression in order to reduce over-diagnosis and over-treatment as a consequence. On the other hand, however, health professionals should consider determining a differential depression diagnosis when depressive symptoms are reported by elderly in order to increase recognition and adequate depression treatment in this age group.

## Additional files

Additional file 1: Proportions of 12-month clinician-diagnosed depression and CIDI-based 12-month major depression (MDD), stratified for time lag. (DOCX 24 kb)
Additional file 2: Socio-demographic and health-related characteristics of participants who report a 12-month clinician-diagnosed depression who did or did not meet the criteria for 12-month major depressive disorder (MDD). (DOCX 25 kb)
Additional file 3: Mental health characteristics of participants who report a 12-month clinician-diagnosed depression who did or did not meet the criteria for 12-month major depressive disorder (MDD). (DOCX 22 kb)

## Abbreviations

CIDI: Composite International Diagnostic Interview
DEGS1: German Health Interview and Examination Survey for Adults
DEGS1-MH: Mental health module of the German Health Interview and Examination Survey for Adults
DSM-IV: Diagnostic and Statistical Manual of Mental Disorders, 4th edition
GNHIES98: German National Health Interview and Examination Survey 1998
MDD: Major depressive disorder
PHQ-9: Patient Health Questionnaire-9
SES: Socio-economic status
