# Supplementary material for: Respondents’ report of a clinician-diagnosed depression in health surveys: comparison with DSM-IV mental disorders in the general adult population in Germany
Source: BMC Psychiatry. 2017 Jan 23;17:39. doi: 10.1186/s12888-017-1203-8 (PMC5259958; doi:10.1186/s12888-017-1203-8)
Supplement: Additional file 1: — Proportions of 12-month clinician-diagnosed depression and CIDI-based 12-month major depression (MDD), stratified for time lag. (DOCX 24 kb) [file 12888_2017_1203_MOESM1_ESM.docx]

**Additional file 1** Proportions of 12-month clinician-diagnosed depression and CIDI-based 12-month major depression (MDD), stratified for time lag

A. Proportions of 12-month major depressive disorder (MDD) among participants with clinician-diagnosed depression in the past 12 months, stratified by time lag

|  |  | |
| --- | --- | --- |
|  | **time lag <= 25 weeks** | **time lag >25 weeks** |
|  | %(w) (95%CI) | %(w) (95%CI) |
| Total | 39.4 (29.7-50.0) | 30.9 (15.8-51.6) |
| Sex | | |
| Men | 40.1 (27.2-54.6) | 39.3 (15.8-69.0) |
| Women | 39.1 (26.8-52.8) | 27.9 (13.0-50.1) |
| Age group (years) | | |
| 18-29 years | 61.8 (33.1-84.2) | 65.4 (26.0-91.0) |
| 30-44 years | 41.6 (18.3-69.4) | 50.0 (9.5-90.6) |
| 45-64 years | 37.3 (24.0-52.9) | 23.2 (11.4-41.4) |
| 65-79 years | 31.3 (18.7-47.5) | 22.3 (4.1-65.8) |

B. Proportions of participants with and without clinician-diagnosed depression among participants with and without major depressive disorder (MDD), stratified by time lag

|  | **time lag <= 25 weeks** | | **time lag >25 weeks** | |
| --- | --- | --- | --- | --- |
|  | **12-month clinician-diagnosed depression in participants with 12-month MDD** | **No 12-month clinician-diagnosed depression in participants without 12-month MDD** | **12-month clinician-diagnosed depression in participants with 12-month MDD** | **No 12-month clinician-diagnosed depression in participants without 12-month MDD** |
|  | %(w) (95%CI) | %(w) (95%CI) | %(w) (95%CI) | %(w) (95%CI) |
| Total | 38.5 (30.0-47.8) | 95.9 (94.6-96.9) | 21.5 (10.7-38.5) | 95.5 (93.6-96.9) |
| Sex | | | | |
| Men | 37.3 (24.9-51.6) | 97.4 (96.3-98.2) | 36.5 (14.8-65.6) | 98.0 (96.0-99.0) |
| Women | 39.2 (27.7-52.0) | 94.4 (91.9-96.1) | 17.9 (8.1-34.9) | 93 (89.3-95.5) |
| Age group (years) | | | | |
| 18-29 years | 31.8 (16.4-52.6) | 98.4 (95.9-99.4) | 11.1 (3.4-30.6) | 99.0 (96.5-99.7) |
| 30-44 years | 21.8 (8.8-44.7) | 97.4 (95.3-98.6) | 17.5 (2.6-63.2) | 98.2 (95.1-99.4) |
| 45-64 years | 53.4 (40.3-66.1) | 94.4 (91.5-96.4) | 38.4 (20.4-60.3) | 91.5 (87.5-94.3) |
| 65-79 years | 47.5 (26.5-69.3) | 94.8 (92.5-96.4) | 30.1 (10.2-61.8) | 95.6 (83.4-99.0) |
