# Supplementary material for: Respondents’ report of a clinician-diagnosed depression in health surveys: comparison with DSM-IV mental disorders in the general adult population in Germany
Source: BMC Psychiatry. 2017 Jan 23;17:39. doi: 10.1186/s12888-017-1203-8 (PMC5259958; doi:10.1186/s12888-017-1203-8)
Supplement: Additional file 2: — Socio-demographic and health-related characteristics of participants who report a 12-month clinician-diagnosed depression who did or did not meet the criteria for 12-month major depressive disorder (MDD). (DOCX 25 kb) [file 12888_2017_1203_MOESM2_ESM.docx]

**Additional file 2** Socio-demographic and health-related characteristics of participants who report a 12-month clinician-diagnosed depression who did or did not meet the criteria for 12-month major depressive disorder (MDD) ^a^

|  | **Clinician-diagnosed depression and MDD**  **(n=96)** | **Clinician-diagnosed depression only**  **(n=153)** | **MDD only (n=188)** | **Non-cases by both instruments**  **(n=3945)** |  |
| --- | --- | --- | --- | --- | --- |
|  | Column %(w) 95%CI | Column %(w) 95%CI | Column %(w) 95%CI | Column %(w) 95%CI | Overall p value |
| Socio-demographic characteristics |  |  |  |  |  |
| Sex |  |  |  |  |  |
| Women | 67.1 (55.9-76.7) | 70.7 (61.0-78.8) | 72.6 (64.1-79.6) | 48.5 (46.4-50.6) | <0.0001 |
| Age group (years) |  |  |  |  |  |
| 18-29 | 18.4 (10.4-30.5) | 6.5 (3.1-13.4) | 30.8 (22.1-41.1) | 19.1 (17.6-20.7) | <0.0001 |
| 30-44 | 18.2 (8.1-36.0) | 13.8 (8.3-22.1) | 35.1 (25.8-45.8) | 25.0 (23.3-26.8) |  |
| 45-64 | 45.9 (34.4-57.9) | 55.2 (44.2-65.7) | 23.2 (17.3-30.5) | 35.6 (33.7-37.5) |  |
| 65-79 | 17.5 (10.0-28.8) | 24.4 (17.0-33.8) | 10.9 (6.6-17.4) | 20.3 (18.9-21.8) |  |
| Married and living with partner or in steady relationship | | | | | |
| Yes | 55.1 (41.6-67.9) | 64.6 (52.3-75.2) | 64.4 (53.9-73.7) | 80.8 (78.9-82.6) | <0.0001 |
| No | 44.9 (32.1-58.4) | 35.4 (24.8-47.7) | 35.6 (26.3-46.1) | 19.2 (17.4-21.1) |  |
| Socio-economic status ^1^ |  |  |  |  |  |
| Low | 32.0 (20.2-46.8) | 32.2 (22.0-44.6) | 24.9 (16.8-35.2) | 17.8 (16.0-19.7) | 0.0024 |
| Middle | 54.1 (39.5-68.0) | 52 (41.1-62.8) | 60 (50.2-69.0) | 61.1 (59.0-63.2) |  |
| High | 13.9 (7.1-25.3) | 15.7 (9.8-24.3) | 15.2 (10.1-22.1) | 21.2 (19.2-23.1) |  |
| Social support ^2^ |  |  |  |  |  |
| Poor | 34.4 (22.9-48.1) | 17.3 (10.7-26.6) | 18 (11.0-28.1) | 10.5 (9.2-11.9) | <0.0001 |
| Moderate | 37 (27.1-48.1) | 50.2 (39.2-61.2) | 46.4 (36.9-56.2) | 49.7 (47.5-51.8) |  |
| Strong | 28.6 (19.0-40.6) | 32.5 (22.5-44.4) | 35.5 (26.8-45.4) | 39.8 (37.6-42.1) |  |
| Employment |  |  |  |  |  |
| Never employed | 12.2 (4.4-29.4) | 8.8 (4.1-17.6) | 2.3 (0.8-6.1) | 3.1 (2.4-4.1) | <0.0001 |
| In employment before | 46.2 (31.8-61.2) | 47.8 (37.0-58.8) | 23.7 (16.4-33.1) | 29.7 (27.8-31.7) |  |
| In employment | 41.6 (28.4-56.2) | 43.5 (33.1-54.5) | 74.0 (64.6-81.6) | 67.1 (65.1-69.1) |  |
| Community size ^3^ |  |  |  |  |  |
| Rural (< 5000 inh.) | 14.9 (6.5-30.9) | 13.8 (7.6-23.8) | 11.3 (6.5-19.1) | 15.5 (10.4-22.6) | 0.0348 |
| Small town (5000-<20,000) | 11.9 (6.2-21.4) | 19.8 (11.2-32.7) | 19.7 (11.7-31.2) | 24.8 (18.5-32.5) |  |
| Mid-sized town (20,000-<100,000) | 28.0 (16.6-43.2) | 22.8 (14.4-34.2) | 35.1 (24.3-47.6) | 29.4 (22.7-37.2) |  |
| Large town (≥100,000) | 45.2 (29.5-61.9) | 43.5 (30.5-57.5) | 33.9 (23.2-46.6) | 30.3 (23.4-38.1) |  |
| Health-related characteristics | | | | | |
| Number of outpatient physician visits (12 months), mean (95%CI) | 24.2 (18.7-29.6) | 17.8 (12.5-23.1) | 8.0 (5.6-10.5) | 7.0 (6.7-7.4) |  |
| Number of chronic somatic conditions ^4^, mean (95%CI) | 1.6 (1.3-2.0) | 1.7 (1.4-2.0) | 0.7 (0.5-0.9) | 1.0 (0.9-1.0) |  |
| SF-36 physical component score (0-100) (norm-based scoring), mean (95%CI) ^5^ | 45.5 (42.6-48.5) | 46.0 (43.6-48.4) | 51.4 (49.6-53.3) | 51.7 (51.3-52.1) |  |
| SF-36 mental component score (0-100) (norm-based scoring) , mean (95%CI) ^5^ | 30.9 (27.5-34.3) | 36.5 (34.6-38.4) | 40.0 (37.6-42.5) | 51.0 (50.6-51.3) |  |

^a^ All characteristics examined were assessed in DEGS1

^1^ Classified as low, medium and high using an index based on information on education, occupational status and net household income.

^2^ Oslo-3 Social Support Scale (OSS-3), self-perceived social support was categorized as poor (3–8 points), moderate (9–11 points) and strong support (12–14 points).

^3^ Based on official administrative municipal codes for the place of residence

^4^ The following somatic diagnosed diseases were included if they were present in the past 12 months: hypertension, diabetes, dyslipidaemia, epilepsy, rheumatoid arthritis, gout, bronchial asthma, hepatitis, gastric-duodenal ulcer, inflammatory bowel disease; the following somatic diagnosed diseases were included if they were ever present: coronary heart disease (CHD) (myocardial infarction or other CHD), stroke, heart failure, chronic renal disease, cirrhosis of the liver, osteoarthritis, osteoporosis, Parkinson's disease, cancer

^5^ Assessed with the SF-36, higher values indicating better health-related quality of life
