# Supplementary material for: Respondents’ report of a clinician-diagnosed depression in health surveys: comparison with DSM-IV mental disorders in the general adult population in Germany
Source: BMC Psychiatry. 2017 Jan 23;17:39. doi: 10.1186/s12888-017-1203-8 (PMC5259958; doi:10.1186/s12888-017-1203-8)
Supplement: Additional file 3: — Mental health characteristics of participants who report a 12-month clinician-diagnosed depression who did or did not meet the criteria for 12-month major depressive disorder (MDD). (DOCX 22 kb) [file 12888_2017_1203_MOESM3_ESM.docx]

**Additional file 3** Mental health characteristics of participants who report a 12-month clinician-diagnosed depression who did or did not meet the criteria for 12-month major depressive disorder (MDD)

|  | **Clinician-diagnosed depression and MDD**  **(N=96)** | **Clinician-diagnosed depression only**  **(N=153)** | **MDD only (N=188)** | **Non-cases by both instruments**  **(N=3945)** |  |
| --- | --- | --- | --- | --- | --- |
|  | Column  %(w) (95%CI) | Column  %(w) (95%CI) | Column  %(w) (95%CI) | Column  %(w) (95%CI) | **p** |
| 12-month mental disorders based on the Composite International Diagnostic Interview (CIDI) ^a^ | | | | | |
| Any anxiety disorder^1^ | 75.9 (63.1-85.3) | 42.1 (31.8-53.1) | 59.9 (50.9-68.3) | 14.5 (13.0-16.2) | <0.0001 |
| Any affective disorder without MDD | 48.6 (35.2-62.2) | 24.8 (17.0-34.7) | 21.3 (14.7-29.7) | 2.6 (2.1-3.4) | <0.0001 |
| Possible psychotic disorder (screening) | 18.9 (8.5-37.1) | 16.3 (9.6-26.4) | 8.4 (4.3-15.7) | 1.6 (1.2-2.3) | <0.0001 |
| Any substance use disorder ^2^ | 18.0 (10.3-29.5) | 13.6 (8.4-21.2) | 10.5 (6.2-17.4) | 5.1 (4.2-6.2) | <0.0001 |
| Any somatoform disorder (pain disorder or SSI4/6)^3^ | 22.8 (14.4-34.2) | 10.0 (5.6-17.0) | 18.3 (11.7-27.5) | 2.2 (1.7-2.9) | <0.0001 |
| Any GMC/substance induced disorder | 13.9 (4.5-35.4) | 4.0 (1.7-9.2) | 1.7 (0.7-4.5) | 0.7 (0.5-1.2) | <0.0001 |
| Any eating disorder ^4^ | 5.9 (1.9-28.7) | 2.0 (0.6-7.0) | 4.0 (1.6-9.2) | 0.7 (0.4-1.1) | 0.0013 |
| Any of the above without MDD | 89.7 (78.7-95.3) | 57.6 (45.7-68.6) | 72.2 (64.3-79.0) | 22.1 (20.3-24.0) | <0.0001 |
| Depression severity based on the Patient Health Questionnaire-9 (PHQ-9) ^b^ | | | | | |
| Current depressive symptoms^5^ | 47.1 (35.5-59.0) | 16.1 (10.0-24.9) | 14.0 (8.6-22.0) | 1.7 (1.1-2.6) | <0.0001 |
| Affirmed suicide item^6^ | 37.2 (25.6-50.5) | 12.5 (6.9-21.4) | 16.6 (10.5-25.0) | 1.8 (1.3-2.3) | <0.0001 |

^a^ Assessed in DEGS1-MH

^b^ Assessed in DEGS1 and DEGS1-MH

^1^ Including panic disorder, agoraphobia, generalized anxiety disorder, social phobia, specific phobias, obsessive-compulsive disorder, PTSD

^2^ Including alcohol and medication abuse and dependence

^3^ Pain disorder and undifferentiated somatoform disorder as measured by the Somatic Symptom Index, SSI4,6

^4^ Including anorexia nervosa, bulimia nervosa, binge eating disorder

^5^ PHQ-9 sum score ≥10 in DEGS1 and in DEGS1-MH

^6^ Affirmed suicide (at least several days) item in DEGS1 and DEGS1-MH
